# Supplementary material for: Directly recruited GATA6 + peritoneal cavity macrophages contribute to the repair of intestinal serosal injury
Source: Nat Commun. 2021 Dec 15;12:7294. doi: 10.1038/s41467-021-27614-9 (PMC8674319; doi:10.1038/s41467-021-27614-9)
Supplement: Supplementary file 1 — Supplementary Information [file 41467_2021_27614_MOESM1_ESM.pdf]

Directly recruited GATA6+ peritoneal cavity macrophages contribute to the repair of intestinal serosal injury

Honda et al.

Supplementary Information

Supplementary Figures 1-11
Supplementary Table 1 and 2

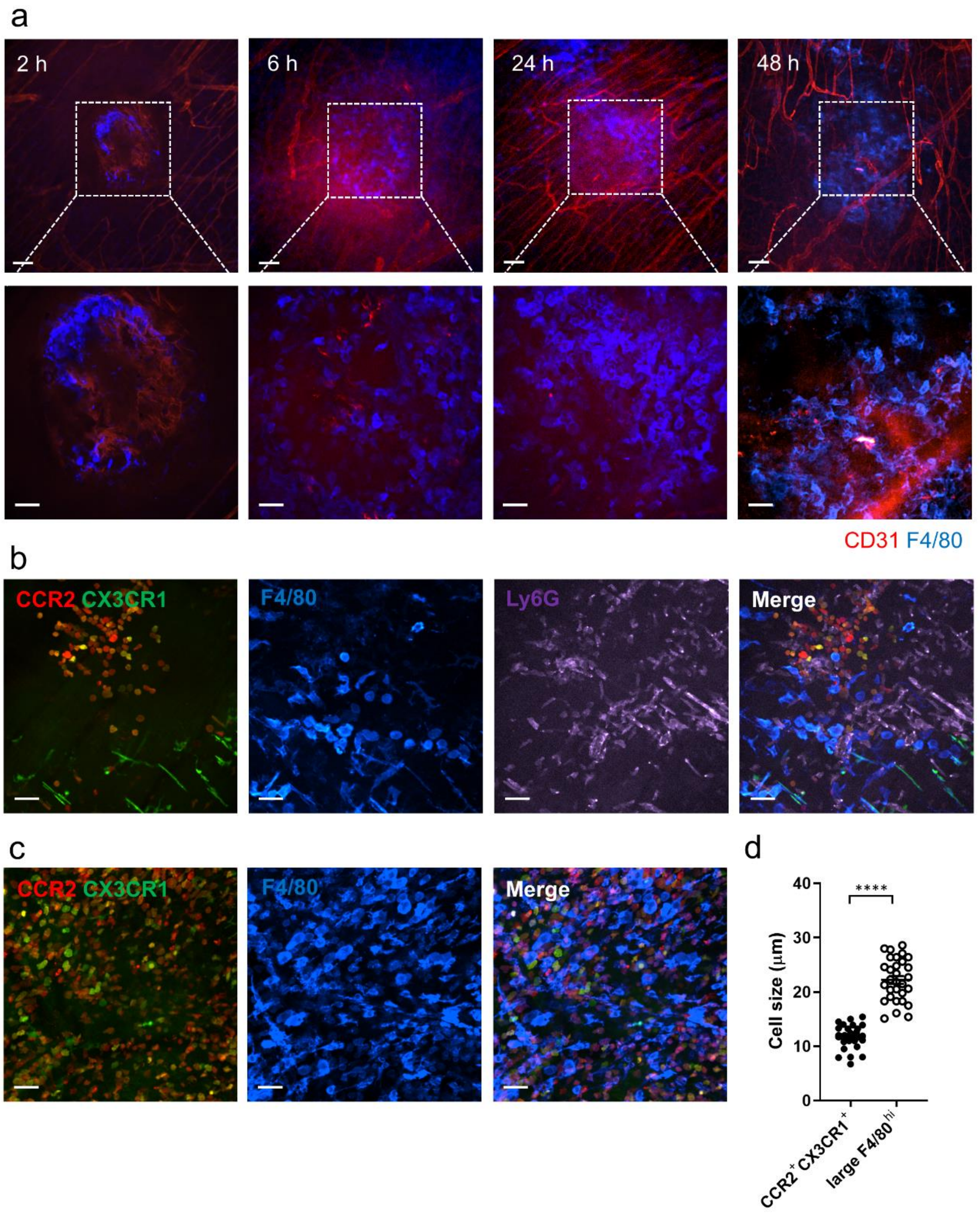

Supplementary Figure 1. Intravital imaging shows the accumulation of large F4/80^{hi} macrophages in the site of intestinal injury. (a) Representative images of the colon at 2, 6, 24 and 48 hrs after focal intestinal injury in C57BL/6 mice. F4/80 antibody (blue) was administered topically to the injury site. Higher magnification of the indicated area is shown in lower row. Scale bars, 100 µm (upper row) and 50 µm (lower row). (b) Representative images of the colon 6 hrs after focal intestinal injury in *Cx3cr1$^{GFP/+}$Ccr2$^{RFP/+}$* mouse. Ly6G antibody (purple) was administered intravenously. Scale bars, 50 µm. (c) Representative images of the colon 24 hrs after focal intestinal injury in *Cx3cr1$^{GFP/+}$Ccr2$^{RFP/+}$* mouse. Scale bars, 50 µm. (d) Quantification of cell size of Cx3cr1$^+$Ccr2$^+$ cells and large F4/80^{hi} cells. n = 30/group (obtained by 3 independent images). Data represent mean ± SEM. ****$p < 0.0001$. *P* values were calculated with two-tailed unpaired Student *t* test (d). Source data are provided as a Source Data file.

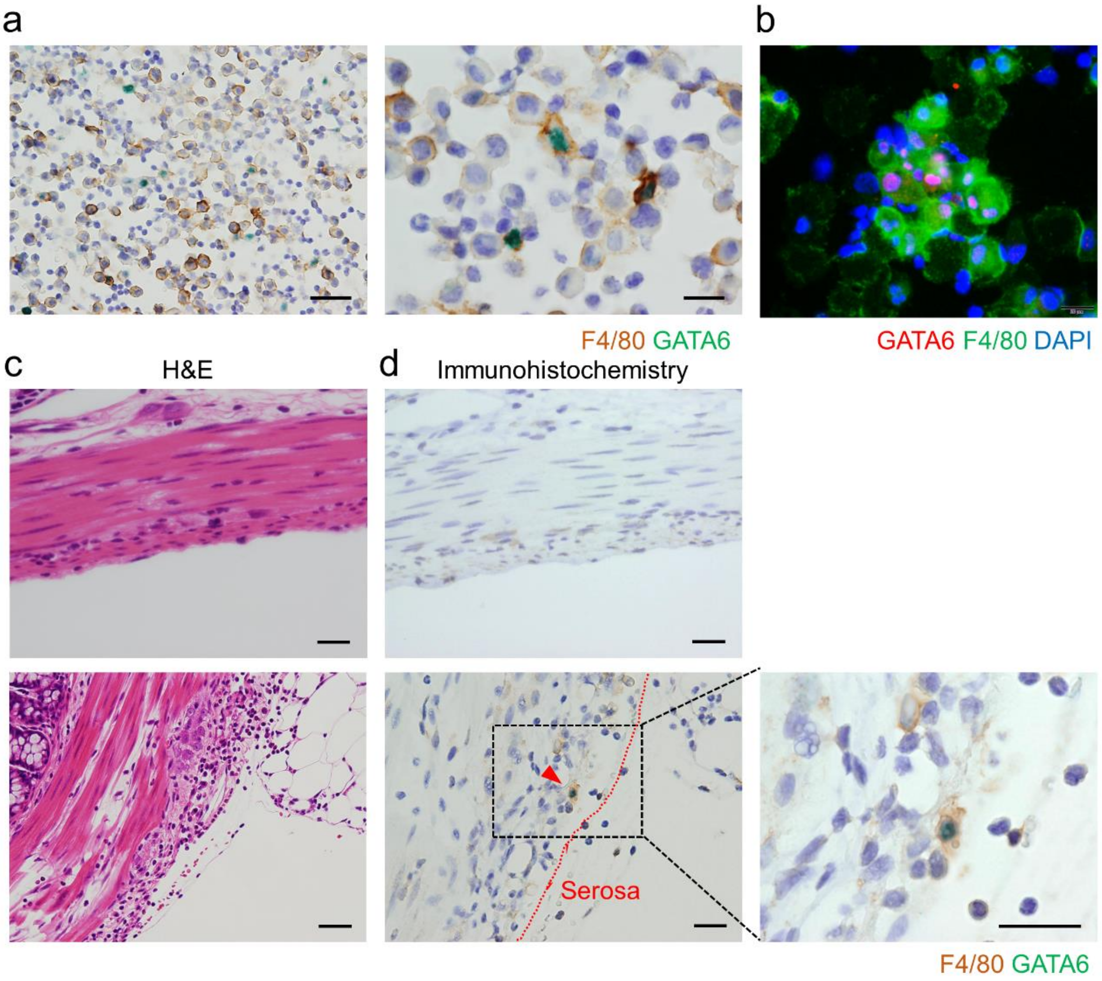

Supplementary Figure 2. GATA6⁺ peritoneal macrophages are accumulating at intestinal injury site. (a) Immunohistochemistry (IHC) staining of formalin fixed paraffin-embedded sample and (b) a cytospin specimen of peritoneal macrophages showed that the GATA6 immunostaining does not overlay with F4/80, but it does overlay with nuclear. Scale bars, 50 μm (left of a), 10 μm (right of a) and 20 μm (b). (c) H&E and (d) immunofluorescence staining for F4/80 (brown) and GATA6 (green) of uninjured or injured colon 24 hrs post-injury (cross section). Arrowhead indicates GATA6⁺ peritoneal macrophage. Scale bars, 20 μm. Data are representative of three independent experiments.

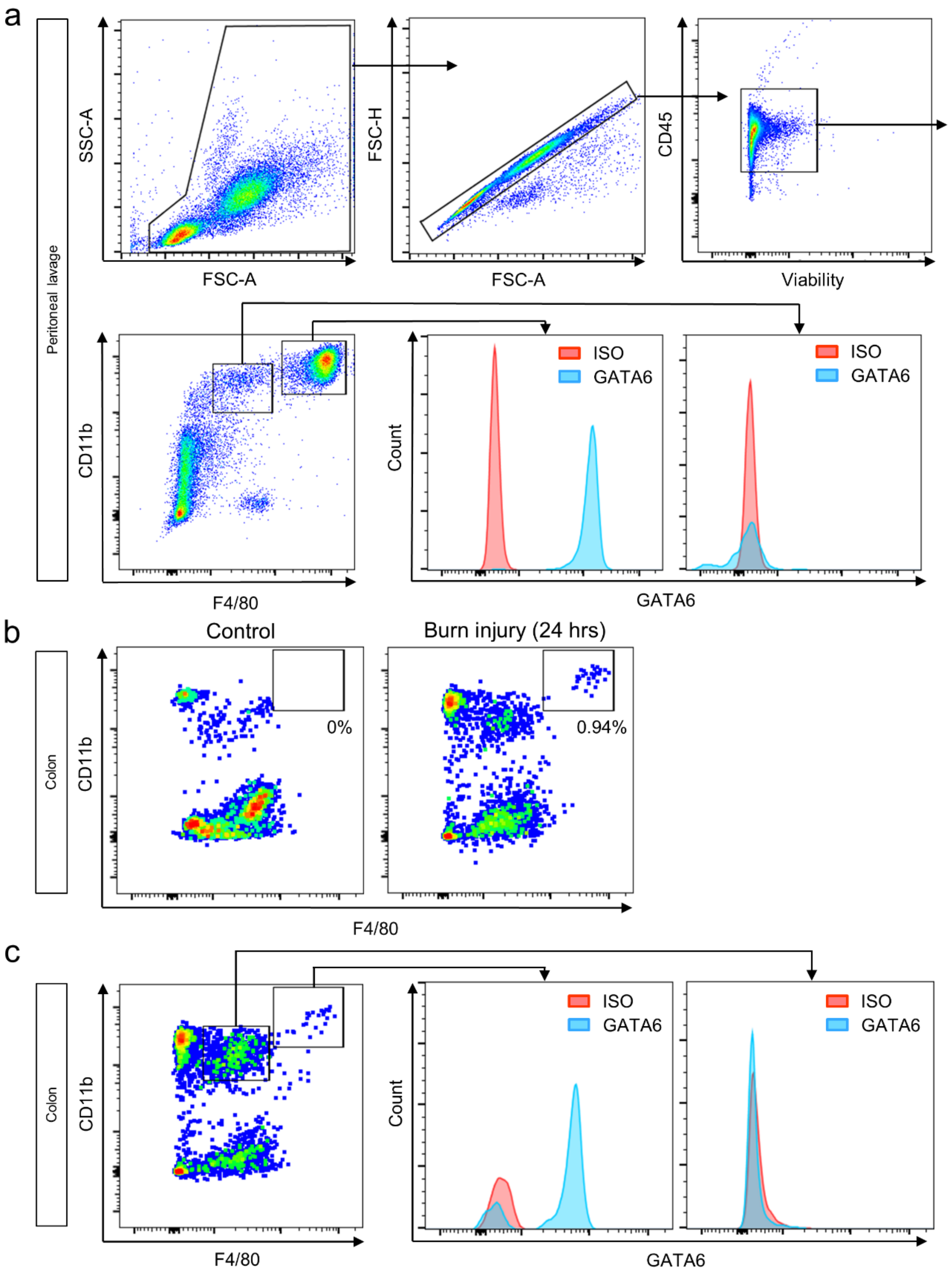

Supplementary Figure 3. Flow cytometry analysis shows that GATA6^{+}CD11b^{hi}F4/80^{hi} peritoneal macrophages accumulate at a sterile intestinal injury site. (a) Flow cytometry sorting strategy for peritoneal macrophages isolated from peritoneal lavage and analysis for GATA6 expression of CD11b^{+}F4/80^{+} macrophages or CD11b^{hi}F4/80^{hi} macrophages. (b) Flow cytometry analysis of colonic macrophages isolated from uninjured or injured colon using biopsy punch at 24 hrs post-injury. Cells were pregated on size, viability and CD45^{+}. (c) Flow cytometry analysis for GATA6 expression of macrophage subsets in injured colon. Cells were pregated on size, viability and CD45^{+}. Data are representative of two independent experiments.

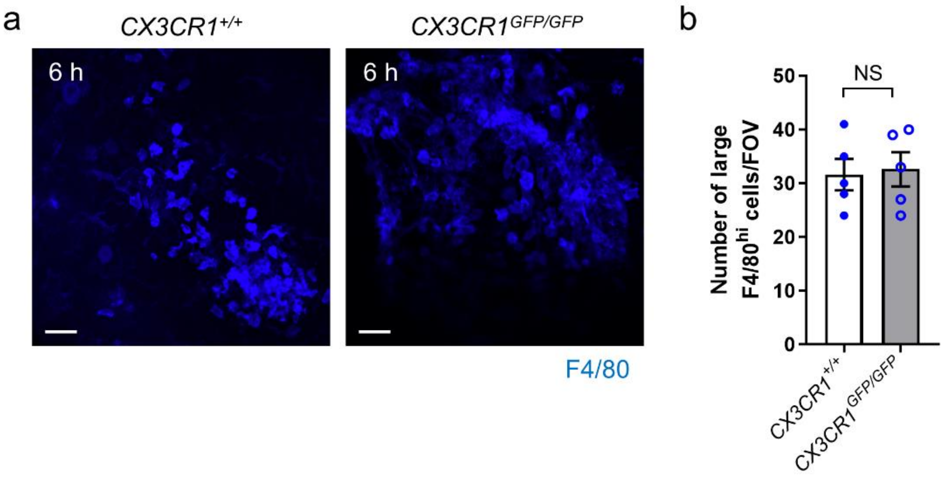

Supplementary Figure 4. Large peritoneal macrophages accumulate to intestinal injury site independent of CX3CR1 ligands. (a) Representative images of the colon 6 hrs after focal intestinal injury in *Cx3cr1*$^{+/+}$ and *Cx3cr1*$^{GFP/GFP}$ (CX3CR1-deficient) mice. F4/80 antibody (blue) was administered topically to the injury site. Scale bars, 50 μm. (b) Quantification of F4/80^{hi} macrophages within intestinal injury site. n = 5/group. Data represent mean ± SEM. NS, not significant. *P* values were calculated with two-tailed unpaired Student *t* test (b). Source data are provided as a Source Data file.

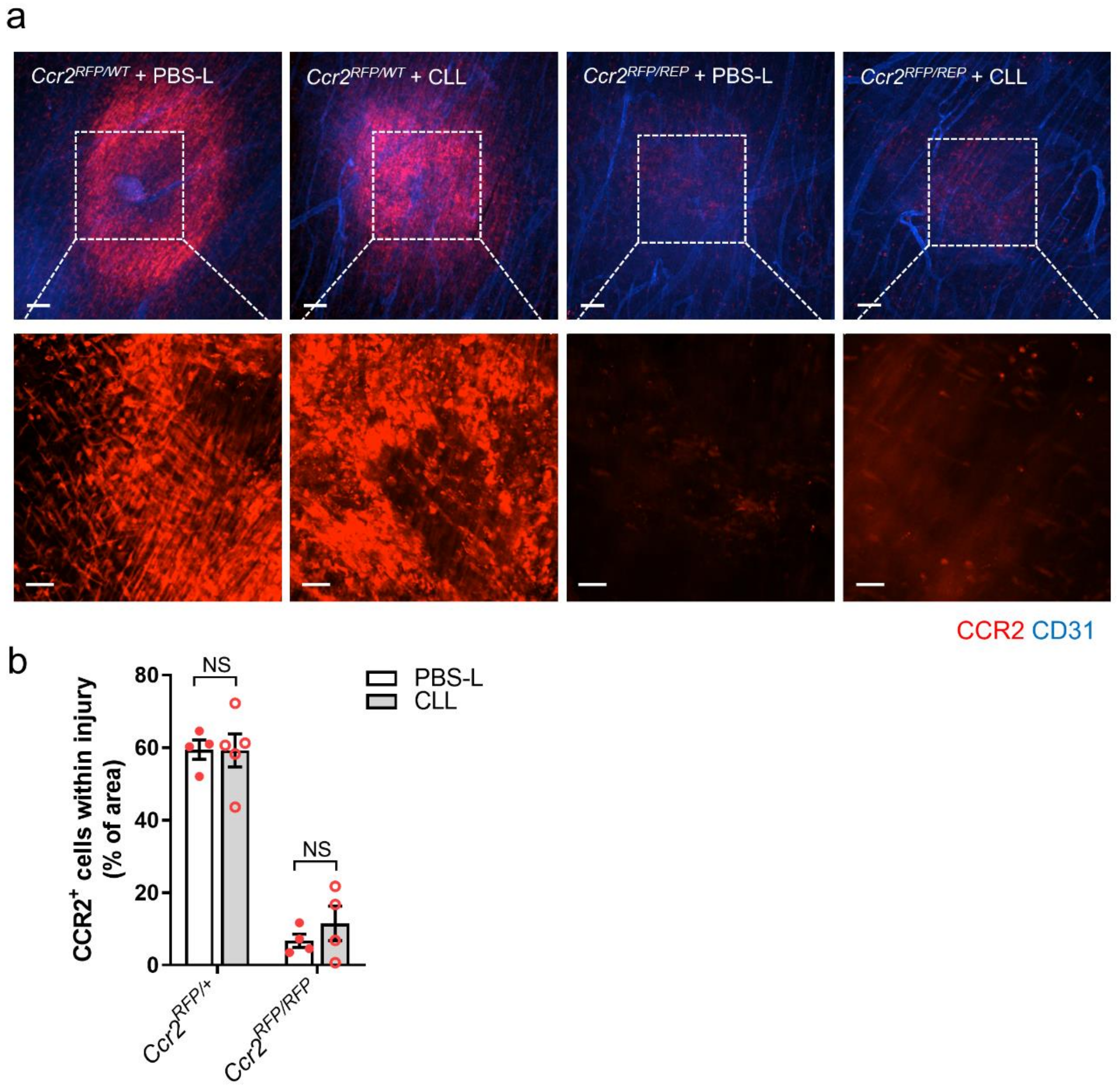

Supplementary Figure 5. Peritoneal administration of CLL does not affect the recruitment of CCR2⁺ monocytes in intestinal injury. (a) Representative images of the colon 48 hrs after focal intestinal injury in *Ccr2*$^{RFP/+}$ and *Ccr2*$^{RFP/RFP}$ (CCR2-deficient) mice. Mice were administered PBS-L or CLL intraperitoneally 4 days before the injury. Scale bars, 100 μm (upper row) and 50 μm (lower row). (b) Quantification of CCR2^{+} cells within intestinal injury site. n = 4 (PBS-L group) and 5 (CLL group). Data represent mean ± SEM. NS, not significant. *P* values were calculated with two-tailed unpaired Student *t* test (b). Source data are provided as a Source Data file.

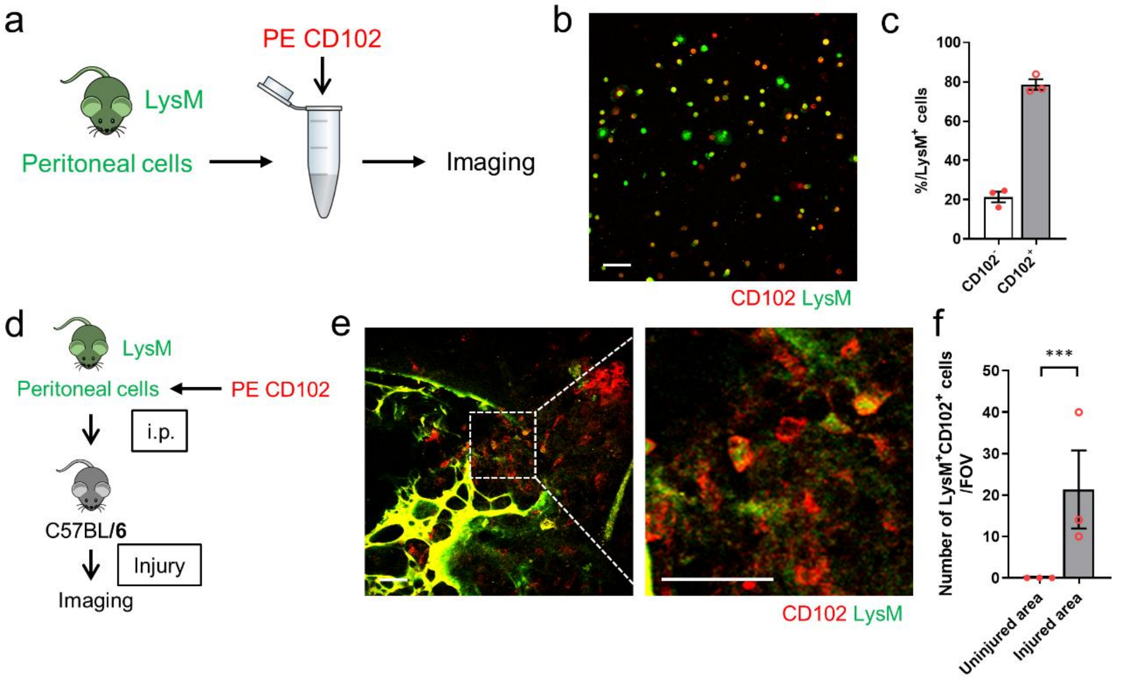

Supplementary Figure 6. ICAM2^{+} large peritoneal macrophages are attracted to the injured colon. (a) Schematic protocol for staining peritoneal cells obtained by LysM-eGFP mice with a PE ICAM2 (CD102) antibody. (b) Representative image and (c) quantification of the LysM^{+}CD102^{+} cells in peritoneal cells. Scale bar, 50 μm. n = 3. (d) Schematic protocol for imaging the colon 6 h post-injury after transferring peritoneal cells obtained by LysM-eGFP mice with PE ICAM2 antibody staining. (e) Representative images and (f) quantification of the LysM^{+}CD102^{+} cells within intestinal injury site. Scale bars, 50 μm. n = 3. Data represent mean ± SEM. ***$p < 0.001$. P values were calculated with two-tailed unpaired Student t test (f). Source data are provided as a Source Data file.

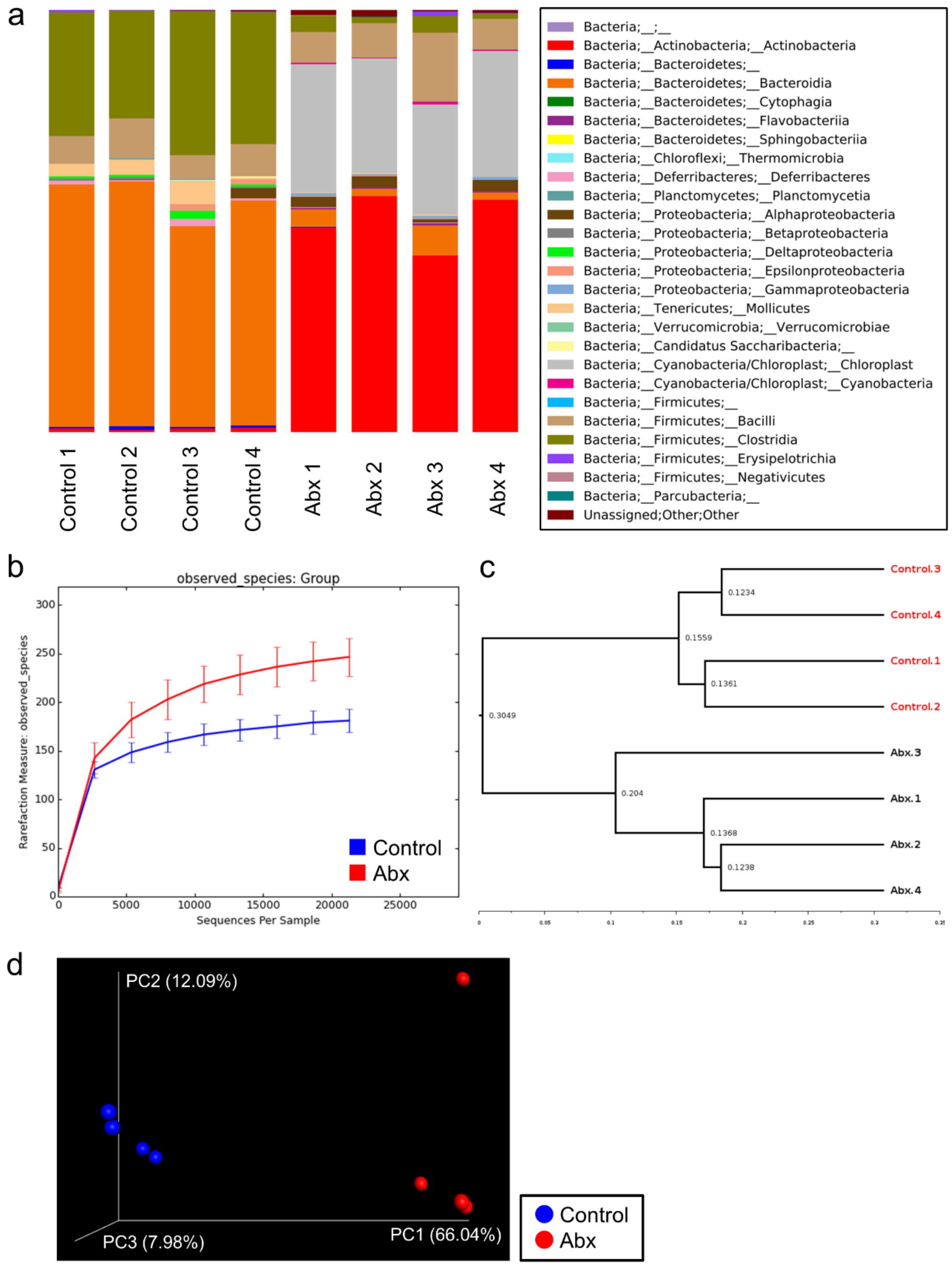

Supplementary Figure 7. Antibiotics use exerts the change of microbial diversity in feces. (a) Relative abundance of bacterial amplicon sequence variants in feces obtained from control or Abx-treated mice. Bar plot is displayed at the class level. n = 4/group. (b) Rarefaction analysis of 16S rRNA gene sequences in fecal microbiota. Each lines represent mean ± SD for each group. n = 4/group. (c) Comparison of the community of gut microbiota in control and Abx-treated mice using the UPGMA algorithm. (d) PCoA of unweighted UniFrac distances of 16S rRNA genes in control and Abx-treated mice.

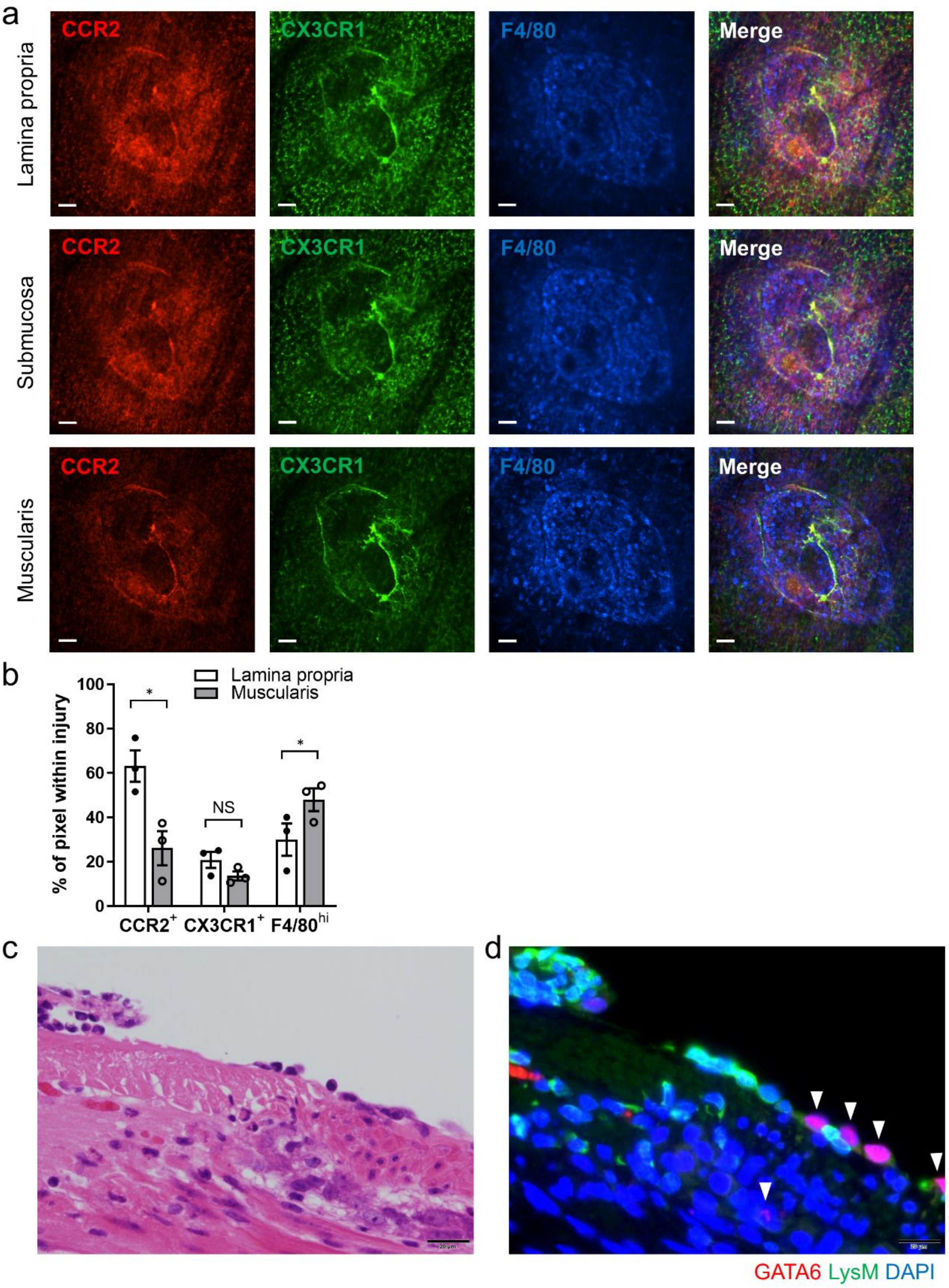

Supplementary Figure 8. $CCR2^+$ monocytes and large F4/80^{hi} peritoneal macrophages accumulate in separate layers of the injured intestine. (a) Representative images of lamina propria, submucosa, and muscularis taken from 24 hrs after focal intestinal injury in *Cx3cr1*$^{GFP/+}$*Ccr2*$^{RFP/+}$ mice. F4/80 antibody (blue) was administered topically to the injury site. Scale bars, 100 μm. (b) Quantification of the proportion of CCR2 (red), CX3CR1 (green) and F4/80^{hi} (blue) pixel within injury site in each layer. n = 3/group. Representative (c) H&E staining of the colon harvested at 24 hrs after injury (cross section) and (d) immunofluorescence staining for GATA6 (red) and DAPI (blue) of injured colon obtained from LysM-eGFP mice. Arrowheads indicate $LysM^+GATA6^+$ peritoneal macrophages. Scale bars, 20 μm. Data represent mean ± SEM. *$p < 0.05$, NS, not significant. *P* values were calculated with two-tailed unpaired Student *t* test (b). Source data are provided as a Source Data file.

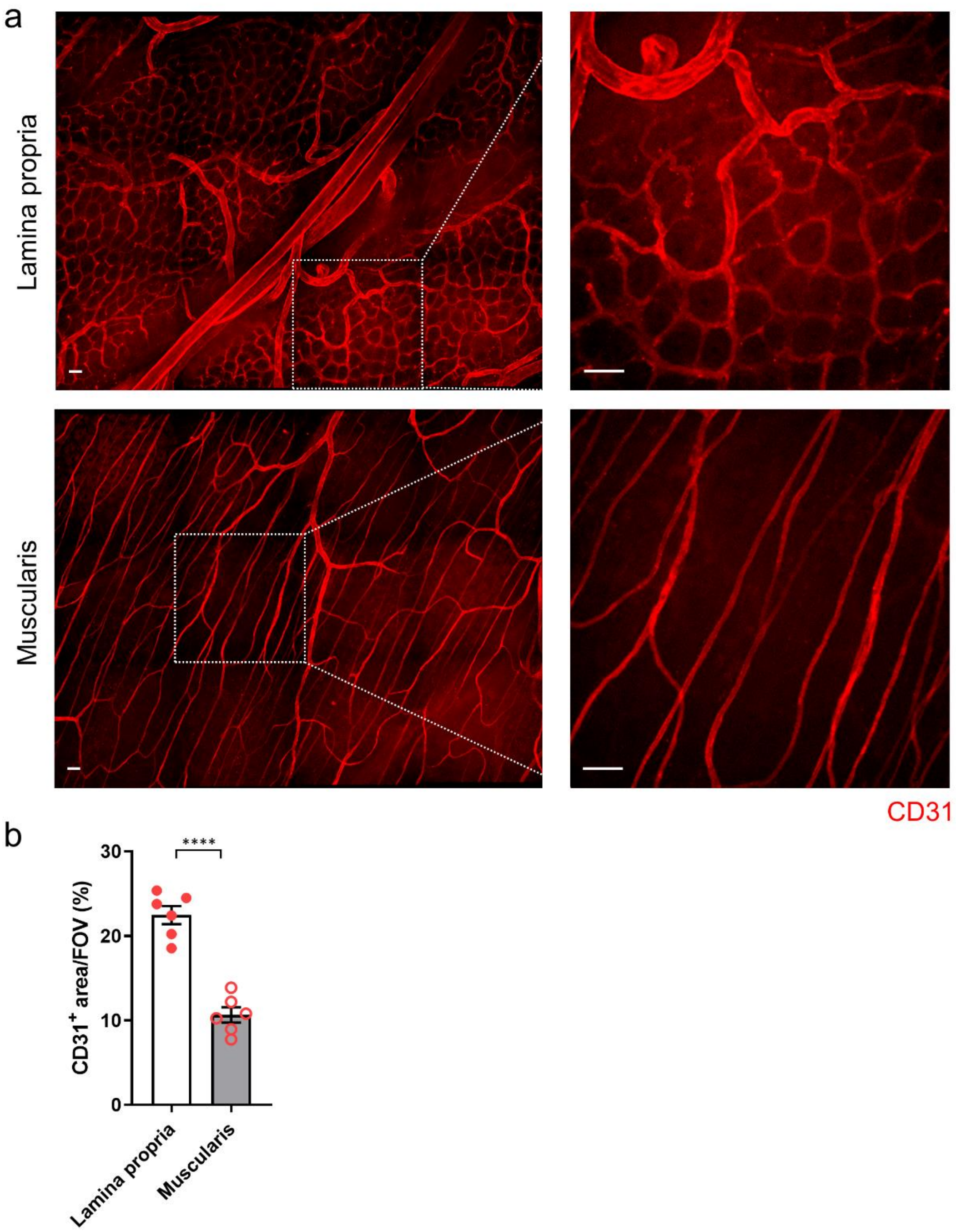

Supplementary Figure 9. Muscularis of the colon has a lower vascular density than the lamina propria. (a) Representative stitch images (from 12 different field of view) of the colonic LP (upper row) and muscularis (lower row) at steady state. Scale bars, 50 μm. (b) Quantification of $CD31^+$ area/field of view (FOV) in each layer. n = 6/group. Data represent mean ± SEM. ****$p < 0.0001$. *P* values were calculated with two-tailed unpaired Student *t* test (b). Source data are provided as a Source Data file.

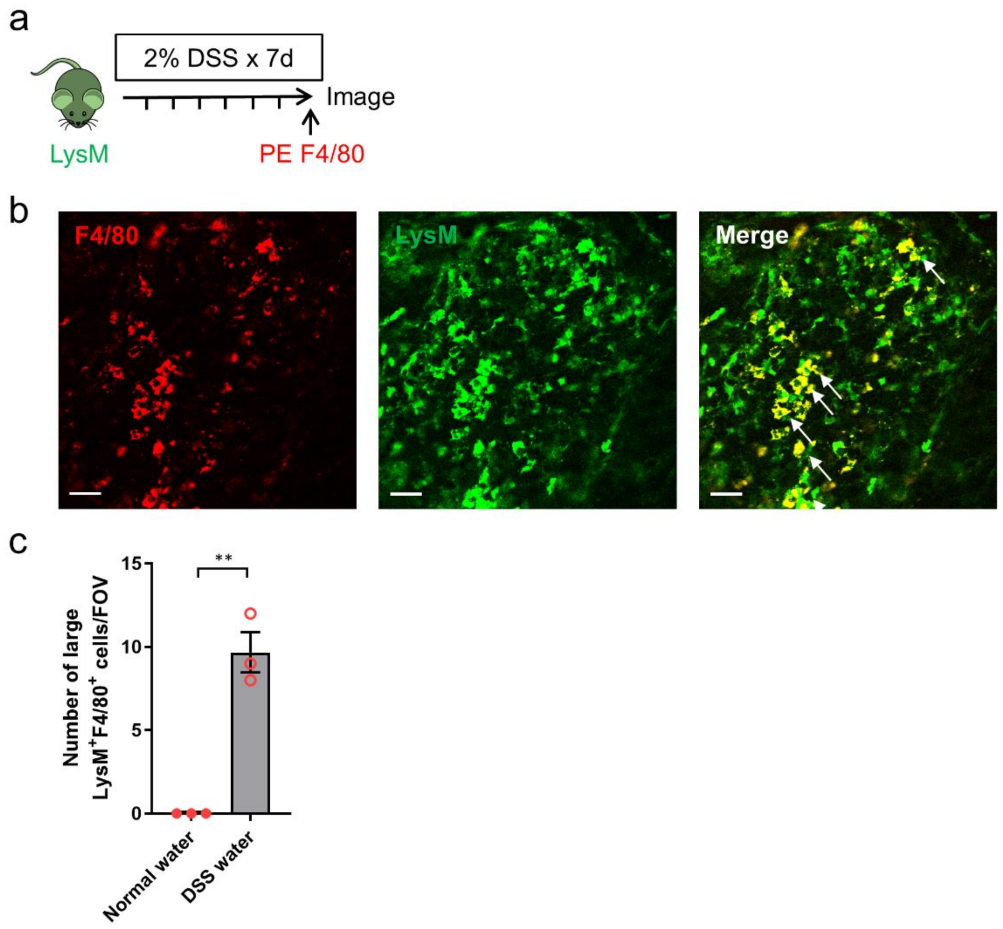

Supplementary Figure 10. Peritoneal macrophages accumulate to the colon in chronic DSS-induced colitis. (a) Schematic protocol for imaging of the colon in chronic DSS-induced colitis. Anti-F4/80 antibody (red) was applied topically before imaging. (b) Representative images of the colonic muscularis in LysM-eGFP mice 7 days after the start of 2% DSS-containing water. Scale bars, 50 μm. (c) Quantification of the number of $LysM^{+}F4/80^{hi}$ cells per FOV in LysM-eGFP mice that were treated with normal water or DSS-containing water. n = 3/group. Data represent mean ± SEM. $^{**}p < 0.01$. *P* values were calculated with two-tailed unpaired Student *t* test (c). Source data are provided as a Source Data file.

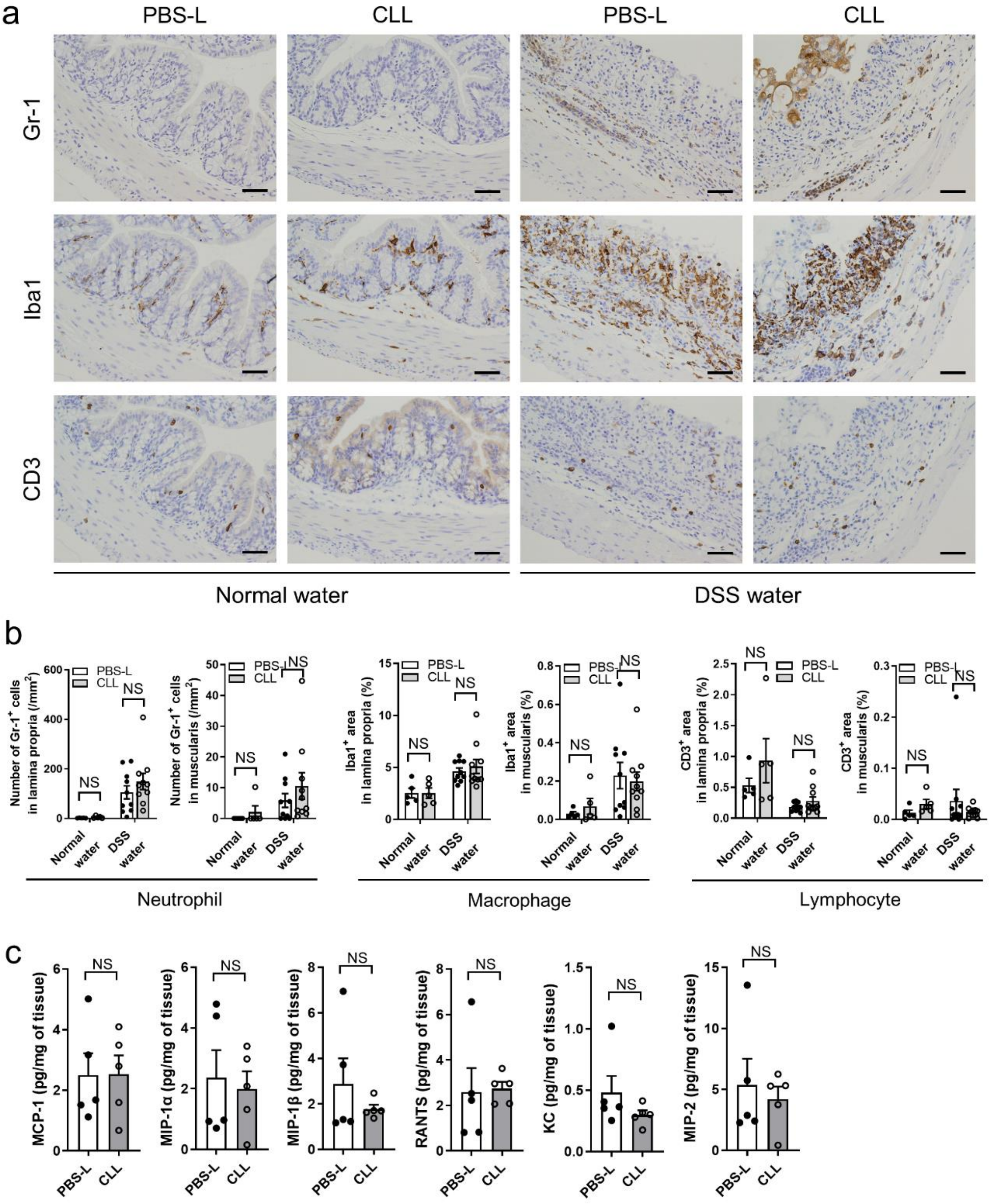

Supplementary Figure 11. Peritoneal macrophages do not play a pivotal role in the recruitment of other immune cells in DSS-induced colitis. (a) Representative IHC images of the colon obtained at 5 d after the start of 4% DSS-containing water and (b) quantification of the degrees of infiltration by neutrophils (Gr-1), macrophages (Iba1), and lymphocytes (CD3) between PBS-L and CLL groups. Scale bars, 100 µm. n = 5 (normal water group) and 10 (DSS water group). (c) Chemokine expression in the colon with or without peritoneal macrophage depletion. n = 5/group. Data represent mean ± SEM. NS, not significant. *P* values were calculated with two-tailed unpaired Student *t* test (b, c). Source data are provided as a Source Data file.

Supplementary Table 1. Disease activity score in 4% DSS colitis

PBS-L

	Day 0	Day 1	Day 2	Day 3	Day 4	Day 5
**General appearance**	0	0	0	0	0	0
**Weight loss**	0	0.4 ± 0.2	0.6 ± 0.2	0.4 ± 0.2	0.2 ± 0.1	1.5 ± 0.2
**Feces consistency**	0	0	0	0	0.2 ± 0.2	0.4 ± 0.3
**Rectal bleeding**	0	0	0	0.8 ± 0.3	2.0 ± 0.0	2.2 ± 0.2
**Total**	0	0.4 ± 0.2	0.6 ± 0.2	1.2 ± 0.2	2.4 ± 0.2	4.1 ± 0.4

CLL

	Day 0	Day 1	Day 2	Day 3	Day 4	Day 5
**General appearance**	0	0	0	0.4 ± 0.2	0.3 ± 0.2	0.3 ± 0.2
**Weight loss**	0	0.4 ± 0.2	0.5 ± 0.2	0.4 ± 0.2	0.9 ± 0.4	2.1 ± 0.4
**Feces consistency**	0	0	0.2 ± 0.2	0.6 ± 0.3	1.0 ± 0.3	1.8 ± 0.5*
**Rectal bleeding**	0	0	1.0 ± 0.5*	2.0 ± 0.4	2.6 ± 0.3	2.4 ± 0.3
**Total**	0	0.4 ± 0.2	1.7 ± 0.6	3.0 ± 0.9	4.8 ± 0.9*	6.6 ± 0.9*

*$p < 0.05$ compared to PBS-L-treated control

P values were calculated with two-tailed Mann-Whitney *U* test.

Supplementary Table 2. Antibodies used for immunocytochemistry (ICC) and immunohistochemistry (IHC)

Primary antibody	Species	Company	Catalog #
CD3	rabbit	Nichirei Biosciences, Inc.	413591
F4/80	rat	Bio-Rad Laboratories, Inc,	MCA497G
GATA6	rabbit	Cell signaling technology, Inc	5851
GR-1	rat	Southern Biotech	1900-01
Biotinylated HABP	human	Hokudo, Co., Ltd.	BC41
IBA-1	rabbit	FUJIFILM Wako Pure Chemical Corp.	019-19741
**Secondary antibody**			
**For ICC**			
Anti-rabbit	goat	Thermo Fisher Scientific	A11012
Anti-rat	goat	Thermo Fisher Scientific	A11006
**For IHC**			
anti-rabbit	goat	Nichirei Biosciences, Inc	424141
anti-rat	goat	Nichirei Biosciences, Inc	414311
PO labeled streptavidin	N/A	Nichirei Biosciences, Inc	426061

CD3, cluster of differentiation 3; GATA6, GATA binding protein 6; GR-1, granulocyte receptor-1 antigen; HABP, hyaluronan binding protein; IBA-1, ionized calcium-binding adapter molecule 1; PO, peroxidase; N/A, not applicable
