# Supplementary material for: Directly recruited GATA6 + peritoneal cavity macrophages contribute to the repair of intestinal serosal injury
Source: Nat Commun. 2021 Dec 15;12:7294. doi: 10.1038/s41467-021-27614-9 (PMC8674319; doi:10.1038/s41467-021-27614-9)
Supplement: Supplementary file 3 — Description of Additional Supplementary Files [file 41467_2021_27614_MOESM3_ESM.pdf]

Description of Additional Supplementary Files

File Name: Supplementary Movie 1

Description: **$CX3CR1^+$ macrophages do not directly infiltrate to the injury site in the colon.** Intravital visualization using SD-IVM shows that $CCR2^+$ monocytes (red) are accumulating in the intestinal injury site (upper right), meanwhile, $CX3CR1^+$ macrophages (green) do not recruit to injury site despite their location. Video was recorded at 6 hrs after burn injury for 20 min using a 10× objective. The elapsed time is displayed in the upper right.
