# Supplementary material for: Directly recruited GATA6 + peritoneal cavity macrophages contribute to the repair of intestinal serosal injury
Source: Nat Commun. 2021 Dec 15;12:7294. doi: 10.1038/s41467-021-27614-9 (PMC8674319; doi:10.1038/s41467-021-27614-9)
Supplement: Supplementary file 5 — Reporting Summary [file 41467_2021_27614_MOESM5_ESM.pdf]

nature research

Corresponding author(s): Masaki Honda

Last updated by author(s): Oct 26, 2021

Reporting Summary

Nature Research wishes to improve the reproducibility of the work that we publish. This form provides structure for consistency and transparency in reporting. For further information on Nature Research policies, see our Editorial Policies and the Editorial Policy Checklist.

Statistics

For all statistical analyses, confirm that the following items are present in the figure legend, table legend, main text, or Methods section.

n/a	Confirmed	
☐	☒	The exact sample size (*n*) for each experimental group/condition, given as a discrete number and unit of measurement
☐	☒	A statement on whether measurements were taken from distinct samples or whether the same sample was measured repeatedly
☐	☒	The statistical test(s) used AND whether they are one- or two-sided<br>*Only common tests should be described solely by name; describe more complex techniques in the Methods section.*
☐	☒	A description of all covariates tested
☐	☒	A description of any assumptions or corrections, such as tests of normality and adjustment for multiple comparisons
☐	☒	A full description of the statistical parameters including central tendency (e.g. means) or other basic estimates (e.g. regression coefficient) AND variation (e.g. standard deviation) or associated estimates of uncertainty (e.g. confidence intervals)
☐	☒	For null hypothesis testing, the test statistic (e.g. *F*, *t*, *r*) with confidence intervals, effect sizes, degrees of freedom and *P* value noted<br>*Give P values as exact values whenever suitable.*
☒	☐	For Bayesian analysis, information on the choice of priors and Markov chain Monte Carlo settings
☒	☐	For hierarchical and complex designs, identification of the appropriate level for tests and full reporting of outcomes
☒	☐	Estimates of effect sizes (e.g. Cohen's *d*, Pearson's *r*), indicating how they were calculated

Our web collection on statistics for biologists contains articles on many of the points above.

Software and code

Policy information about availability of computer code

Data collection	Volocity software 6.1 (PerkinElmer) was used to drive the confocal microscope and for 3D rendering, acquisition, and analysis of images. DNA was extracted from fecal pellet using a QIAamp Fast DNA Stool Mini Kit (QIAGEN) and 16S rRNA genes were amplified and sequenced using an Illumina MiSeq (Illumina, San Diego, CA, U.S.A.).
Data analysis	Flow cytometry data were analyzed using FlowJo v10 software (Tree Star). Imaging data were analyzed using Image J v1.45 software package (NIH). Chemokine data were obtained using Luminex assay and analyzed with StarStation V.2.3 (Applied Cytometry System). The 16S rRNA operational taxonomic units (OTUs) were selected from the combined reads using a de-novo OTU picking protocol clustered at 97% identity using the Quantitative Insights Into Microbial Ecology (QIIME) pipeline software. Number of OTUs, Chao 1, and Shannon were calculated, and rarefaction curve was generated using QIIME. Statistical analyses were performed using GraphPad Prism v8.0 software (GraphPad Software Inc.).

For manuscripts utilizing custom algorithms or software that are central to the research but not yet described in published literature, software must be made available to editors and reviewers. We strongly encourage code deposition in a community repository (e.g. GitHub). See the Nature Research guidelines for submitting code & software for further information.

Data

Policy information about availability of data

All manuscripts must include a data availability statement. This statement should provide the following information, where applicable:
- Accession codes, unique identifiers, or web links for publicly available datasets
- A list of figures that have associated raw data
- A description of any restrictions on data availability

The authors declare that the data supporting the findings of this study are available within the paper and its supplementary information files. We deposited the

sequencing data to NCBI Genbank and provided the accession code to the 'Data Availability' section. All other data that support the findings of this manuscript are available from the corresponding author upon reasonable request.

Field-specific reporting

Please select the one below that is the best fit for your research. If you are not sure, read the appropriate sections before making your selection.

☒ Life sciences ☐ Behavioural & social sciences ☐ Ecological, evolutionary & environmental sciences

For a reference copy of the document with all sections, see nature.com/documents/nr-reporting-summary-flat.pdf

Life sciences study design

All studies must disclose on these points even when the disclosure is negative.

Sample size	Sample sizes were chosen based on previous experiments to enable statistical analyses such as standard deviation and t-tests (Honda et al., Nat Commun. 2020 Mar 12;11(1):1329.).
Data exclusions	No data were excluded.
Replication	Experiments were reliably reproduced. Experimental findings were reproduced at least twice to ensure a consistence.
Randomization	Mice of the same age/sex were randomly allocated to each experimental groups.
Blinding	Imaging data acquisition and analyses were not blinded but all assays were performed at the same time for all groups of a given experiment. Since all conditions were subjected to the same analyses, blinding was not considered to be necessary. Investigators were blinded in pathological analysis.

Reporting for specific materials, systems and methods

We require information from authors about some types of materials, experimental systems and methods used in many studies. Here, indicate whether each material, system or method listed is relevant to your study. If you are not sure if a list item applies to your research, read the appropriate section before selecting a response.

Materials & experimental systems

n/a	Involved in the study	
☐	☒	Antibodies
☒	☐	Eukaryotic cell lines
☒	☐	Palaeontology and archaeology
☐	☒	Animals and other organisms
☒	☐	Human research participants
☒	☐	Clinical data
☒	☐	Dual use research of concern

Methods

n/a	Involved in the study	
☒	☐	ChIP-seq
☐	☒	Flow cytometry
☒	☐	MRI-based neuroimaging

Antibodies

Antibodies used	Antibodies against CD11b (#17-0112-82; M1/70), CD31 (#12-0311-82; PECAM-1, 390), CD45 (#45-0451-82; 30-F11), F4/80 (#12-4801-82; BM8) were obtained from eBioscience. Antibodies against Ly6G (#127612; 1A8) were obtained from Biolegend. Antibody against CD44 (#553134; IM7) was obtained from BD Biosciences. Antibody against GATA6 (#26452; D61E4) was obtained from Cell Signaling Technology. Anti-CD16/32 antibody (#BE0307; 2.4G2 clone) was obtained from Bio X Cell. Anti-CD3 antibody (#413591; SP7) was obtained from Nichirei Biosciences, Inc. Anti-GR1 antibody (#1900-01; RB6-8C5) was obtained from Southern Biotech. Anti-IBA-1 antibody (#019-19741) was obtained from FUJIFILM Wako Pure Chemical Corp.
Validation	The dose of antibody used in this study was determined based on previous experiments conducted (Honda et al., Nat Commun. 2020 Mar 12;11(1):1329.).

Animals and other organisms

Policy information about studies involving animals; ARRIVE guidelines recommended for reporting animal research

Laboratory animals	C57BL/6 mice (#000664), LysM-eGFP mice, Cx3cr1GFP/+ (knock-in) mice, Cx3cr1GFP/GFP (CX3CR1-deficient) mice (#005582), and Nr4a1-/- mice (#006187) were obtained from The Jackson Laboratory. Generation of Ccr2RFP/RFP (CCR2-deficient) and Ccr2RFP/+ (knock-in) mice have been previously described. Cx3cr1GFP/+Ccr2RFP/+ mice were generated by crossing Cx3cr1GFP/GFPCcr2RFP/RFP mice with C57BL/6 mice. Mice of 8-12 weeks of age were used for experiments. Mice were gender matched for experiments and experimental/control mice were bred separately.

Wild animals: The study does not involve wild animals.

Field-collected samples: The study does not involve samples collected from the field.

Ethics oversight: All experiments were performed according to guidelines of the Institutional Animal Committee of Kumamoto University.

Note that full information on the approval of the study protocol must also be provided in the manuscript.

Flow Cytometry

Plots

Confirm that:

☒ The axis labels state the marker and fluorochrome used (e.g. CD4-FITC).

☒ The axis scales are clearly visible. Include numbers along axes only for bottom left plot of group (a 'group' is an analysis of identical markers).

☒ All plots are contour plots with outliers or pseudocolor plots.

☒ A numerical value for number of cells or percentage (with statistics) is provided.

Methodology

Sample preparation: Cells were isolated from colon. Residual red blood cells were lysed using ACK lysing buffer (Invitrogen). Colonic cells were isolated as described in methods. The cells were blocked using anti-CD16/32 antibody (2.4G2 clone; Bio X Cell) for 30 min. Then, cells were stained for 30 min with antibodies for specified markers.

Instrument: FACSCanto and LSR-II (BD Biosciences)

Software: FlowJo v10 software (Tree Star)

Cell population abundance: Sorting was not performed.

Gating strategy: Cell debris were first excluded by SSC-A and FSC-A. Then, doublets were excluded using single-cell gating based on FSC-H and FSC-A. CD45 and viability dye staining were used to gate the live leukocytes. Further gating strategies depend on the experimental setup.

☒ Tick this box to confirm that a figure exemplifying the gating strategy is provided in the Supplementary Information.
